# Effects of Changed Aircraft Noise Exposure on Experiential Qualities of Outdoor Recreational Areas

**DOI:** 10.3390/ijerph7103739

**Published:** 2010-10-20

**Authors:** Norun Hjertager Krog, Bo Engdahl, Kristian Tambs

**Affiliations:** 1 Department of Air Pollution and Noise, Division of Environmental Health, Norwegian Institute of Public Health, PO Box 4404 Nydalen, N-0403 Oslo, Norway; 2 Division of Mental Health, Norwegian Institute of Public Health, PO Box 4404 Nydalen, N-0403 Oslo, Norway; E-Mails: bo.engdahl@fhi.no (B.E.); kristian.tambs@fhi.no (K.T.)

**Keywords:** aircraft noise, annoyance, area problems, panel study, outdoor recreation, experiential effects, telephone survey

## Abstract

The literature indicates that sound and visual stimuli interact in the impression of landscapes. This paper examines the relationship between annoyance with sound from aircraft and annoyance with other area problems (e.g., careless bicycle riding, crowding, *etc.*), and how changes in noise exposure influence the perceived overall recreational quality of outdoor recreational areas. A panel study (telephone interviews) conducted before and after the relocation of Norway’s main airport in 1998 examined effects of decreased or increased noise exposure in nearby recreational areas (*n* = 591/455). Sound from aircraft annoyed the largest proportion of recreationists, except near the old airport after the change. The decrease in annoyance with sound from aircraft was accompanied by significant decreases in annoyance with most of the other area problems. Near the new airport annoyance with most factors beside sound from aircraft increased slightly, but not significantly. A relationship between aircraft noise annoyance and perceived overall recreational quality of the areas was found.

## 1. Introduction

Most studies within the area of noise research have dealt with the impact of noise in residential areas. But the last decades there has been increasing awareness of the importance of protecting the special sound qualities of natural areas for the benefit of outdoor recreationists [1–4]. While natural sounds are experienced positively even at loud levels, technological sounds are generally experienced negatively in natural settings [5–7]. To escape from noise, and to experience the silence and peace of nature have been found to be among the most important reasons for visiting outdoor recreational areas [8,9]. In studies that examine the impact of noise together with other potential area problems or disturbances, noise has been found to be among the most salient problems [10–12].

The present paper presents results from a panel study, conducted as telephone interviews in connection with the relocation of Norway’s main airport in 1998. The old airport was totally closed down, while the new main airport was an existing airport that was expanded. A special issue of importance regarding the effects of an airport relocation is how the changes in aircraft noise exposure affect the experience in local outdoor recreational areas. While some studies have examined responses to various types of aircraft overflights in national parks and wilderness areas [11–21], knowledge about effects in local urban or rural recreational areas are lacking, and especially knowledge about the effects of abrupt changes in noise exposure. The effect of the airport change on noise annoyance during single visits to nearby outdoor recreational areas was examined by cross sectional field studies that combined survey data with noise measurements [22,23]. The panel studies that are presented here examine the more lasting impression of experiences in the study areas after a season of use before and after the airport change. To the best of our knowledge, this is the first panel study to examine experiential effects of both a decrease and an increase in overall aircraft noise exposure in outdoor recreational areas in the vicinity of commercial airports.

Commonly, the experiential effect of changes in noise exposure is assumed to be predictable on the grounds of dose-response relationships derived from data collected in a stable state situation [13,17,24,25]. However, there are some indications from studies in residential areas that especially an abrupt change in noise exposure levels may cause people to “overreact” compared to the predictions made by steady-state dose-response relationships [26–34]. The field data from the same recreational areas before and after the airport change were analyzed to test the influence of the situation of change on the dose-response relationship in an outdoor recreational setting [22,23]. A strong effect of the situation of change was found, beyond what would be expected from data from before the moving of the airport. Several explanations of the change effect have been suggested in the literature on noise effects in residential areas [34]. One possible explanation is that attitudes modifying the exposure-response relationship changes. For instance, overall opinion of the neighbourhood could change [34]. The “overreaction” effect in terms of noise annoyance in the recreational areas might also indicate that the changes in noise exposure levels affect a broader set of experiential dimensions than “noise annoyance” alone. The main purpose of this paper is to examine how changed aircraft noise exposure possibly influences the experience of other area conditions, as well as the perceived overall recreational quality of outdoor recreational areas.

### 1.1. Review of the Literature

There are some indications in the literature that noise may affect a broader range of experiential dimensions. Perhaps most obviously, noise may interfere with the natural quiet of a site [11,13,19]. But noise may also influence other aspects of the recreational experience, and detract from the experience and enjoyment of the visitors [11,21,35].

An interaction effect of image and sound has been demonstrated in the perception of the general quality of landscapes. In an experimental study, Carles *et al.* [36] let the subjects evaluate the pleasantness of six images and six sounds alone and in combination. The images were natural or semi-natural scenes, and urban parks. The sounds ranged from purely natural sounds to mechanical sounds caused by human activity. The evaluation of each stimulus (visual or aural) was found to be modified by the co-presence of another stimulus. The situations that were rated most positively were those where there was coherence between the visual and the aural stimulus. In a postal survey to visitors of wilderness areas Tarrant *et al.* [14] found that overflights influenced visitor solitude and tranquillity more than annoyance. The effect measures were related to the single aircraft overflight that the visitors best remembered. Anderson *et al.* [5] utilized three different experimental procedures for the assessment of the impact of different sounds on the aesthetic evaluation of outdoor settings. The subjects were either evaluating the sounds in a field setting, or both setting and sounds were described verbally, or they were presented to photographs and tape recordings. All three procedures produced similar results. While natural and animal sounds were found to enhance the evaluation of natural sites, technological sounds were found to detract from the evaluation of the sites. In another laboratory experiment Mace *et al.* [37] examined the effect of helicopter noise on the evaluation of a natural vista. Slides were presented together with either 40 dB(A) or 80 dB(A) helicopter noise. These conditions were compared to a control condition where background natural sounds accompanied the slides. The presence of helicopter noise was found to adversely affect all dimensions that were evaluated in the study, which were: naturalness, preference, scenic beauty, freedom, annoyance, solitude, and tranquillity. An effect was found on all measures for both noise exposure levels, but the effect was most pronounced at the highest noise level. A comparison of the affect states before and after the experimental condition showed that positive affect states decreased while negative affect states increased significantly. The findings are mainly confirmed by later research [38,39]. An experimental study examining the evaluation of different combinations of natural scenes and sounds found that the evaluations in terms of pleasantness primarily were differentiated by the sounds that accompanied the scenes, while there was little differentiation in the evaluation of the visual impressions [40]. The effect, and the direction of the effect, may depend on the context, urban or natural, and basic expectations to sound and environmental qualities in the different settings [5,41].

An interaction effect of visual and aural stimuli has also been indicated in the urban context. However, where the residential or urban setting is the basis for study, the influence of vision on sound perception, not the other way around, has mostly been focused. The opposite focus and findings in regard to the direction of the effect in studies of natural versus urban settings presumably are related to basic differences in setting functions, and what may be expected of sound, silence, and dominance of built or natural visual elements. In the natural setting, technological sounds are assumed to potentially adversely affect visual qualities, and thereby recreational benefits of the natural areas. A stress reducing effect of viewing nature is well established in the literature [42–44], while noise is an ambient stressor that possibly might detract from this effect [37]. On the other hand, in the predominantly built environment of the urban setting, natural landscape elements might reduce the stress caused by noise, compared to settings without natural elements. While most studies have focused on the positive effects of viewing nature, a recent experimental study also indicates that nature sounds facilitate recovery from stress [45].

The first to explore the relationship between the visual and aural perception of the city was Southworth [46]. Not focusing on noise, but on the general influence of sounds on the perception of the visual city, Southworth found that the aural and visual sensations mutually influenced each other in the impression of a particular site. He took subjects on trips around the city of Boston, and let them describe their experience in their own words. The subjects could either hear, but not see, or see, but not hear, or both see and hear. In regard to the perception of noise in the residential setting, the visual street aesthetic has been found to influence the degree of noise annoyance [47–49]. People living in streets that were rated more highly on visual aesthetic quality (e.g., pretty/ugly, aesthetic architectonic appearance, greens) were found to be less annoyed at the same noise levels than people living in streets that were rated lower on visual dimensions. In a series of five experimental studies and two field surveys Tamura *et al.* [50] examined the audiovisual interactions in the formation of annoyance of urban places. The results indicated that annoyance was based on a combination of auditory and visual conditions. First, the degree of tree plantations was found to reduce annoyance with traffic noise, and thereby annoyance of a space, or “the personal impression of feeling uncomfortable within the place”, as stated by Tamura *et al.* [50]. On the other hand, there were also indications that the presence of plants might “awaken the feeling of annoyance” by the larger expectations to quiet that the plantation arouse. Another experimental study found that the influence of the visual context on the sound ratings depended on the type of sounds [41]. The evaluations of bird song and traffic were negatively influenced by increasing degrees of urbanization in the visual stimuli. The ratings of human sounds did not depend on the context. In the experimental study of Anderson *et al.* [5] cited above both natural and technological sounds were relatively neutral in regard to the aesthetic evaluation of the most urban settings. An effect was found when the visual stimuli contained natural elements. Although not consistent on all points, all the above cited studies point to an interaction effect between the perception of visual and sound stimuli, at least in some contexts and for some types of sound. The findings may be explained in terms of cognitive consistency theories [51]. According to these theories, people tend to seek internal coherence in their evaluations of the various components of a situation. Simon *et al.* [51] studied experimentally what happens in the process of reaching a verdict. They found that not only do various components influence the experience of the whole. In the process, the general impression also influences the perception of the parts. Cognitive consistency theories would predict that people would align their evaluations of different conditions to conform to a consistent representation of an outdoor recreational area. But also, that a general impression of change, to the better or the worse, could influence how various area conditions are perceived.

Aircraft noise is but one of several environmental factors that may detract from the recreational experience in outdoor recreational areas. Other environmental factors that have been found to be potential problems in outdoor recreational areas are for instance crowding, litter, damage to natural, historical, or cultural resources, development, and maintenance of facilities [11,12]. These potential “annoyances” are usually examined item by item. However, on the background of previous findings and notions from cognitive consistency theories we propose that they may also be examined as visual or aural stimuli that might interact in their influence on the recreational experience. That is, the existence and perception of one factor might influence how the other is perceived. This would also mean that a change in noise exposure levels would influence a broader range of experiences than noise annoyance alone.

### 1.2. Objectives and Research Questions

It was aimed at putting the issue of aircraft noise into perspective with other potential area problems, and to study how changes in noise levels affected the perception of these other environmental factors, and the overall qualities of the outdoor recreational areas. More specifically, these questions were examined:

1. How annoying is the sound from aircraft compared to other potentially annoying aspects of the environment in the areas before and after the change?
2. To what degree does the level of annoyance with aircraft noise change following the changes in aircraft noise levels?
3. Does the perception of other potentially adverse environmental factors change following the changes in aircraft noise levels?
4. How is the perceived overall recreational quality of the areas affected by the changes in aircraft noise levels?

## 2. Method

### 2.1. Study Areas

**Figure 1.**
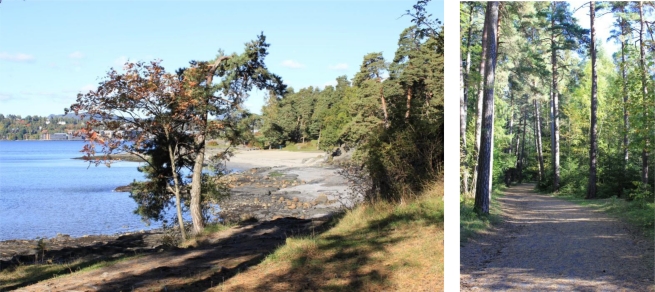
Photos from Bygdøy.

**Figure 2.**
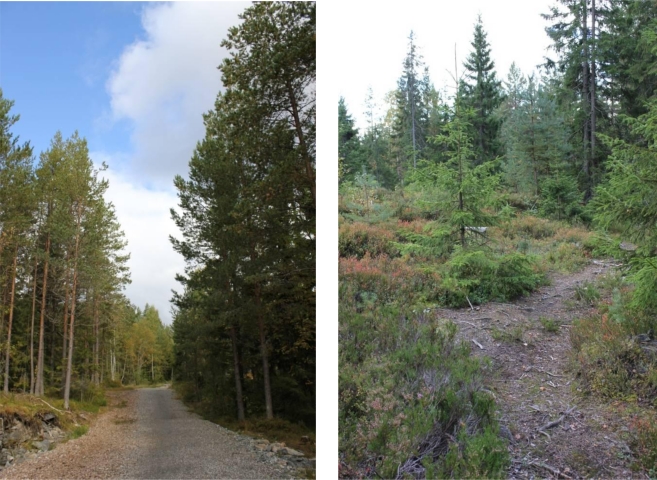
Photos from Romeriksåsen.

The areas studied were Bygdøy, near the old main airport, and Romeriksåsen, near the new main airport. Figure 1 and Figure 2 show photos to illustrate the types of recreational areas that were studied. The areas were selected on the grounds of their location relative to the airports, and because they were much used by the local communities. Bygdøy is situated just outside the City of Oslo at the Oslo fjord. The area contains a popular beach and a small forest. Bygdøy is a relatively small recreational area of about 2.6 km^2^. Romeriksåsen is situated in the countryside, about 19 miles outside Oslo. Romeriksåsen is a large forested area, about 7,600 km^2^. There are several small lakes in the area, and a network of forest roads and paths. There are also private and public cabins.

For practical reasons, it was not possible to obtain data on the respondents’ individual sound exposure during an entire season of use. We find it useful, however, for the generalization of the results to give a qualified description of what exposure levels, and changes in exposure levels that were typically experienced in the areas. Average exposure levels for visitors on single trips to the areas were obtained from field studies with other respondents conducted in 1998 and 1999 [22,23,52]. Field studies were conducted at Bygdøy and in Romeriksåsen at the same time of the year that was focused on in the present study. At Bygdøy, the variation in aircraft noise levels was moderate across the area because of its limited size, and it was judged as sufficient to conduct sound recordings at one site.

In Romeriksåsen, field studies were carried out at three different sites. It should be noted that we do not have measurements from the northernmost part of the area. The data collection was done over five to ten weekend days for each sub-study. The results are summarized in Table 1. There was a marked shift in mean exposure levels at Bygdøy, both measured in A-weighted equivalent aircraft sound levels (Aircraft L_Aeq_), in proportion of the time that sound from aircraft exceeded 55 dB, and in the proportion of the time aircraft could be heard. Regarding Romeriksåsen, the mean individual exposure in terms of equivalent aircraft sound level increased by less than three dB at the measurement sites. But the proportion of the time aircraft could be heard was more than doubled after the change, which indicates a substantial increase in the amount of aircraft overflights, especially at low levels. Except for some cutting at Bygdøy, other area conditions were not assessed to actually change.

### 2.2. Procedure

Telephone interviews with a panel of respondents were conducted before and after the change of airport location. That is, the same samples were interviewed before and after the change. To avoid sensitizing people toward aircraft noise during the first interview, the study was masked as a general study about outdoor recreational areas in the region. The real purpose was not revealed until the end of the second interview.

The sampling and the telephone interviewing were conducted by Opinion A/S, an established and reputable commercial opinion poll company. Initially, gross samples of random telephone numbers were drawn from postal addresses adjacent, or close to each of the recreational areas. Within each household, the person over 18 years who last had a birthday was selected. Only people who had visited the area at least once during the past three months, and at least two times during the past six months (Romeriksåsen), or two times during the past 12 months (Bygdøy) were interviewed as visitors to the area. The additional criteria to number of visits, beyond the three months period, were set to exclude the completely casual visitors. We were primarily interested in examining the effects of the airport change on the residents who potentially would use the area repeatedly as a local recreational resource. The reason for the extension of the period for Bygdøy is that only three additional months would cover midwinter. During winter there is relatively little outdoor activity at Bygdøy.

Both times the respondents were asked to recall experiences in the area during the past three months prior to the interview. The period focused on was the peak season in each area for hiking and related activities. Since changes in experiences were to be examined, it was considered of great importance to keep the season fixed for the initial study and the follow-up of the same area. Seasonal differences in meteorology influence both the transmission of sound and the context in which the sound is perceived. There will also be seasonal variations in activities and expectations of experiences that might influence the perception of aircraft sound.

The first interviews about Romeriksåsen were conducted in November 1997, and the first interviews about Bygdøy from the middle of May to the middle of June 1998. In 1999, the study was repeated for each area during the same weeks of the year. We will use the term t_1_ (time 1) whenever referring to data from the before situation in either area, and t_2_ (time 2) whenever referring to the after situation. At t_1_ 1,600 visitors were interviewed about Bygdøy, and 1,620 about Romeriksåsen. At the end of the first interview the respondents were asked if they agreed to be contacted again in one year (Bygdøy) or two years (Romeriksåsen).

To actually be interviewed twice as a visitor of the area, the requirements for number of visits should also be met at the time of the second survey. Because Romeriksåsen is a large area with varying degrees of exposure to aircraft noise, a further restriction was made on what experiences to compare. The visitors were categorized according to what part (or parts) of the area they had visited: the northern, middle or the southern part. Only recreationists who visited the same part of the area both years were included in the analyses in this article, to exclude the possibility that changes in experiences between t_1_ and t_2_ were due to the respondents being at different places the two years. These samples consisted of 591 (Bygdøy) and 455 (Romeriksåsen) respondents, respectively.

### 2.3. Dropouts

Forty-five percent of the original Bygdøy sample and 41 percent of the original Romeriksåsen sample could not be interviewed at t_2_. The level of refusal of further participation was, however, not high. Sixteen percent in the original Bygdøy sample and seven percent in the Romeriksåsen sample refused to let their name be recorded at t_1_. At t_2_ four percent of the remaining Bygdøy sample and seven percent of the remaining Romeriksåsen sample refused being interviewed again. The rest of the drop-out was respondents who were not reached at t_2_. The visitors at t_1_ who dropped out of the study (regardless of reason) did not differ systematically from the remaining subjects regarding the central variable of annoyance with aircraft noise.

The demographic composition of the analyzed samples was quite similar to the composition of some field study samples from the same areas and time periods [22,23], which indicates that the samples are representative of the area users. The variables compared were gender, age, and educational level.

### 2.4. Variables and Analyses

The analyses were conducted using the statistical package SPSS 9.0 for Windows. Annoyance with sound from aircraft and with other potentially adverse environmental factors was measured by the respondents’ answers to the question: “If you again think of the past three months. Have you been annoyed by any of the following factors when you have been at Bygdøy/in Romeriksåsen?” The factors were (the longest variable descriptions are shortened in Figures 1 and 2; the full wordings of the shortened variable labels are given in parentheses here): sound from aircraft, careless bicycle riding, crowding, road traffic noise (sound from traffic on nearby road), dogs, vehicles on forest roads, human encroachment on the forest, seeing aircraft, (sound from) power saw or forestry machinery (only in the questionnaire about Romeriksåsen), and shooting (at nearby rifle range/shooting gallery; only in the questionnaire about Romeriksåsen). The response categories were “not annoyed”, “slightly annoyed”, “rather annoyed” or “very annoyed.” The word “sound”, not “noise”, was deliberately used in the wording of the question, to not influence the respondents’ answers by using a word with a negative meaning. The factors beside sound from aircraft were chosen on the grounds of what were expected to be potential problems in the particular study areas. The annoyance variables were dichotomized between slightly and rather annoyed for the analyses. The proportions in the highest annoyance category were compared. The category “rather or very annoyed” signifies that people were more than casually affected. The dichotomization is similar to the one used in studies from the National Parks in the US, except that a five-point scale was used in those studies [13,17,19]. The scale used in the National Park studies was “not at all”, “slightly”, “moderately”, “very much”, or “extremely” annoyed, and the scale was dichotomized between slightly and moderately annoyed [13,17,19]. A four-point scale was used in our study because telephone interviews require that no more alternatives are presented than the respondents can keep in memory.

To compare the proportions annoyed by aircraft noise to the proportions annoyed by other area factors at each point in time, 95% confidence intervals for the proportions “rather or very annoyed” were calculated by the adjusted Wald method. To test whether the proportions annoyed by the sound from aircraft and other environmental factors significantly changed following the changes in aircraft noise exposure, the McNemar test [53] was used. The McNemar test is a non-parametric test for two related dichotomous variables, especially suited for testing changes in response following an intervention. The test utilizes the chi-square distribution to test for differences in distributions.

Perceived changes in recreational quality were measured by asking the respondents to evaluate the quality of the area as an outdoor recreational area, compared to how it was a couple of years ago. The possible answers were “better”, “worse”, “both better and worse”, “no difference” or “not sure”. In addition, the respondents answering “better”, “worse”, or “both better and worse” were asked an open-ended question about the reasons why they felt the area was better or worse than it used to be. The relationship between perceived changes in recreational quality at t_2_ and the changed noise exposure levels was examined in two ways: first, by simple frequency analyses of the proportions answering “aircraft noise” to the open question about reasons for changed quality; second, by crosstable analyses of the relationship between aircraft noise annoyance and perceived improvement (Bygdøy), or deterioration (Romeriksåsen) of recreational quality. Dichotomized versions of both variables were used in the crosstable analyses. Since an improvement in area quality was expected, the categories “better” and “both better and worse” were combined and contrasted to the other categories combined in the analysis of the Bygdøy data. Regarding Romeriksåsen, the categories “worse” and “both better and worse” were combined and contrasted to a combination of the other categories, since a deterioration of area quality was expected.

## 3. Results

### 3.1. Annoyance with Sound from Aircraft Compared to Annoyance with Other Factors

**Figure 3.**
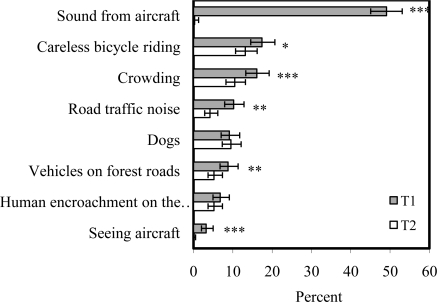
Proportions rather or very annoyed by various environmental factors at Bygdøy, t_1_ and t_2_, with 95% confidence intervals. McNemar test: * −*p* < 0.05; ** −*p* < 0.01; *** −*p* < 0.001 (*n* = 591, visitors both years).

Figure 3 shows the proportions of the respondents visiting Bygdøy (near the old airport) both years who were “rather or very annoyed” by sound from aircraft and other environmental factors. The factors are arranged in descending order after proportions annoyed at t_1_. At t_1,_ sound from aircraft was the potentially adverse environmental factor that annoyed the largest proportion of the recreationists. About half the visitors, 49.1 (95% confidence intervals: 45.1–53.1) percent were “rather” or “very annoyed” by the sound from aircraft. The 2nd highest proportion annoyed by any other factor was 17.4 (14.6–20.7) percent, attributed to careless bicycle riding. Seeing aircraft was the least annoying of the environmental factors, with only 3.2 (2.0–5.0) percent rather or very annoyed. The ranking of factors by the proportions who were rather or very annoyed applies to the sample. The overlap in 95% confidence intervals shows that the total ranking cannot be generalized. At t_2_ almost none, 0.3 (0–1.3) percent of the same respondents were rather or very annoyed by sound from aircraft, and no one was annoyed by seeing aircraft. In addition, sound from road traffic was perceived as less of a problem relative to the other environmental factors at t_2_, and occupies the third to last position in the rank order.

**Figure 4.**
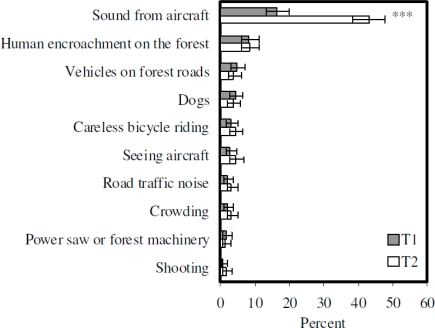
Proportions rather or very annoyed by various environmental factors in Romeriksåsen, t_1_ and t_2_, with 95% confidence intervals. McNemar test: *** −*p* < 0.001 (*n* = 455, visitors to the same part of Romeriksåsen both years).

Figure 4 illustrates the proportions of visitors to the same part of Romeriksåsen (near the new airport) both years, who were “rather or very annoyed” by sound from aircraft and by other potentially annoying environmental factors. At t_1_, sound from aircraft was the environmental factor that annoyed the largest proportion of visitors with 16.3 (13.2–20.0) percent rather or very annoyed. Few recreationists were rather or very annoyed by seeing aircraft, 2.6 (1.5–4.6) percent. Of the other factors, only human encroachment on the forest with 8.1 (5.9–11.0) percent was rather or very annoying to more than five percent of the recreationists. The low percentages rather or very annoyed by most of the factors are more striking than any differences, and make a ranking of the factors less relevant. The overlap in the confidence intervals of the different factors means that the differences cannot be generalized. However, for the purpose of comparison, Figure 4 shows the variables in ranked order. At t_2_, 43.1 (38.6–47.7) percent were rather or very annoyed by sound from aircraft, and 4.2 (2.7–6.5) percent were rather or very annoyed by seeing aircraft. The proportions annoyed by any of the other factors were overall small, like they were at t_1_, and the ranking cannot be generalized.

### 3.2. Changes in Annoyance with Sound from Aircraft and Other Environmental Factors

The asterisks in Figure 3 identify significant differences in the proportions of respondents rather or very annoyed by the various environmental factors between t_1_ and t_2_ in the area where the aircraft noise exposure decreased (Bygdøy). The large decrease in the proportions rather or very annoyed by sound from aircraft was, not surprisingly, highly significant (McNemar test, chi-square 284.031, *p* < 0.001). Also the change from a few percent to no one annoyed by seeing aircraft was significant (*p* < 0.001).

In addition, there was a significant decrease (*p* < 0.001–*p* < 0.05) in the proportions rather or very annoyed by four of the six environmental factors not related to aircraft. One of the small insignificant differences (in annoyance with “human encroachment on the forest”) follows the same trend of decrease, while there is a very slight increase in the proportion annoyed by dogs.

In Romeriksåsen (Figure 4), where the aircraft noise exposure increased, the only significant difference in annoyance between t_1_ and t_2_ was the increase in the proportion rather or very annoyed by sound from aircraft (chi-square 90.377, p < 0.001). Although very slight and not significant, there are increased proportions rather or very annoyed by six of the nine other factors, including seeing aircraft.

### 3.3. Effects of Changed Noise Exposure on the Overall Recreational Quality of the Area

Table 2 shows the visitors’ perception of the overall recreational quality of the areas at t_2_, compared to how it was a couple of years ago. The largest proportion of visitors to Bygdøy experienced that the recreational quality was improved. Although more than 1/3 found that the area had deteriorated, the largest proportion of visitors to Romeriksåsen perceived no difference.

Only 20 percent (*n* = 309) of the recreationists who evaluated Bygdøy as better, or in some aspects better, answered the open question about why they felt the area was better at t_2_. The changes related to the airport were mentioned by 18 percent (*n* = 309) as a reason why the area had become a better place for outdoor recreation. Two percent did not explicitly relate the improvement to the closing of the airport.

All of the respondents who found that Romeriksåsen had deteriorated (*n* = 187) attributed this to some aspect of the airport expansion, and 80 percent mentioned explicitly noise as the reason why the area was not as good for outdoor recreation as earlier. In addition, 26 percent mentioned other reasons why the area was not as good as it used to be.

The results of the crosstabulation analyses of the relationship between aircraft noise annoyance and perceived improvement (Bygdøy), or deterioration (Romeriksåsen) of recreational quality are shown in Table 3 and Table 4. There was a clear relationship between indicating that Bygdøy had become, at least partly, a better outdoor recreational area at t_2_, and being “rather or very annoyed” by aircraft noise at t_1_. Although the difference was highly significant, the proportion that felt that the area was better was also relatively high among those who were “not or slightly annoyed” by sound from aircraft at t_1_.

There was a strong significant relationship between finding that Romeriksåsen had deteriorated, and being rather or very annoyed by aircraft noise at t_2_ (Table 4).

## 4. Discussion

### 4.1. Summary

Sound from aircraft was the environmental factor that annoyed the largest proportion of visitors in all situations, except for the situation in the area near the old airport after it was closed. The proportions of visitors annoyed by other environmental factors were higher in the smaller urban forest area at Bygdøy than in the vast forest of Romeriksåsen, where the proportions annoyed by other factors overall were low. The proportions annoyed by seeing aircraft were low compared to the proportions annoyed by the sound from aircraft.

In both areas there was a large change in annoyance with sound from aircraft after the main airport was moved. In the area near the old airport the decreased noise exposure was followed by a significant decrease in annoyance with most of the other environmental factors examined. There were no corresponding significant increases in annoyance with other factors in the area near the new airport, although there was a tendency toward slightly increased proportions being rather or very annoyed at t_2_. Both the decrease and the increase in noise exposure influenced the perception of the overall recreational quality of the areas at t_2_.

### 4.2. Validity of the Data

Because of the use of panel interviews, we can exclude some of the problems we would have met in concluding from results based on different samples at t_1_ and t_2_. The differences in experience between t_1_ and t_2_ cannot be due to differences between samples. To let the same people evaluate different recreational settings in order to determine situational effects is recommended by Stewart and Cole [54]. Stewart and Cole point to the problem in recreational research of large individual differences that blur situational effects. Except from the unison lack of annoyance with sound from aircraft in the area near the old airport at t_2_, the individual variation in reactions is visible in our data for each point in time. But in comparing t_1_ and t_2_, what varies are the situational conditions, the individuals are the same. Stewart and Cole recommend a diary method instead of global measures gathered some time after the recreational experience in question. In this study, however, we were not interested in the immediate reaction, but the more lasting impression over some time. A reason for this focus was that we were also interested in examining behavioural effects of the changes in noise exposure [55].The recalled impression of the recreational area was assumed to be the basis of later decisions to revisit or not. Since there were no significant differences in annoyance with sound from aircraft at t_1_ between the dropouts and the remaining samples, we assume that the drop-out was not of decisive importance for the results.

A possible disadvantage of a panel study is that the respondents get sensitized toward the special issues of the study during the first interview, which might influence their responses the second time. However, special care was taken not to sensitize the respondents toward possible noise problems or other area problems through the presentation and design of the questionnaire. The study was presented as a study about outdoor recreation in the Oslo region. The questionnaire contained only one question about annoyance with sound from aircraft in the area. The questionnaire contained questions about various aspects of the outdoor recreational experience and use of the area, and was overall not especially problem focused. Evidence from other studies indicate that survey-resurvey bias is not a problem in regard to repeat measures of annoyance responses [34]. All in all, the data are assumed to be a reasonably valid expression of experiential effects of changes in aircraft noise exposure.

In generalizing from the results, some cautions should be kept in mind, however. The effects observed may depend on conditions like the special aircraft noise levels in the areas relative to the existence of other impacts, the amount of change in noise exposure, and the respondents’ level of experience with the area.

### 4.3. Substantial Findings

Aircraft noise was compared to other potential adverse area conditions to examine its relative importance, and to establish a basis for the comparison between the two situations, before and after the airport change. The existence and dominance of different area problems may vary from area to area, or site to site. Some problems may also tend to be more notable than others, given that they are present. Compared to other sound exposures in natural areas, technological sounds have been shown to be perceived most negatively [5–7,35]. It has been suggested that just noticing sound from aircraft may detract from the outdoor recreational experience, because the natural soundscape, free from the sounds of civilization, is an important part of the experience that is sought [11,37]. The finding that sound from aircraft was the environmental factor that annoyed the largest proportion of visitors in most situations is in accordance with findings in the few studies that have compared recreationists’ concern with aircraft noise and other types of area problems [11,12].

An exception was the situation in the area near the old airport after the change, where almost no one was annoyed by the sound from aircraft. This does not mean that aircraft could not be heard in the area after the change. The actual aircraft noise exposure of the respondents in this study is unknown. But Table 1 indicates that the aircraft noise exposure near the old airport actually was quite comparable to that experienced at the field study sites near the new main airport after the change. The largest difference was in the proportion of time aircraft can be heard, which was about half the time near the old airport compared to at the sites near the new airport. The sound exposure levels (Aircraft L_Aeq_, proportion of time over 55 dB) were about the same, or slightly higher at the old airport. The large divergence in reactions to comparable noise levels is similar to the large change effect that was found in dose-response studies from the same study areas [22,23]. The fact that similar results were obtained by different methodology (panel study versus different samples at t_1_ and t_2_) further supports the validity of the data.

The absence of annoyance with aircraft noise near the old airport at t_2_ points to the influence of contextual variables on the perception of sound. Annoyance may be influenced by the visitors’ past experience in the area [13], and the direction of change. One suggested explanation of the change effect is that people get adapted to their present noise environment and judge new noise situations on the basis of this standard [34,56]. Thus, reactions to the same noise situation may be totally different depending on the adaptation level. Our findings fit with the notion of different frames of reference influencing the judgement of similar exposure. However, adaptation-level theory also implies that people chronically exposed to high noise levels become desensitized [34,56]. On this grounds Brown and van Kamp [34] discarded the adaptation-level explanation. They found that it was not in accordance with results from studies with data from both before and after a change. In our study, the large proportion of visitors to Bygdøy that was annoyed by aircraft noise before the change does not support the thesis of desensitization. Further, adaptation-level theory would imply that the change effect is transient, people eventually adapting to their new exposure level. We were not able to study adaptation to change over time, since we only have data from one point in time within one year after the change. However, results from other studies do not support the notion of adaptation to change [34].

Another explanation where the notion of different frames of reference in different noise situations is crucial is the differential response criteria explanation. It suggests that people apply different response criteria and use annoyance rating scales differently in different noise situations [34]. The change effect is explained as a kind of measurement error. People experiencing a change in noise conditions expand their scaling of the noise effects. Thus, their rating of the same noise effects becomes different from those chronically exposed to the same noise levels. The effects are not different; the difference is the use of the scale. We cannot rule out the differential response criteria explanation as an explanation of the “overreaction” to change effect that we found in terms of aircraft noise annoyance. However, the systematic change in annoyance with other area factors at Bygdøy cannot be explained by differential response criteria. This explanation presupposes that there had been a change in exposure to each of the other factors, but this was not the case. The change in annoyance with other factors and the effect on overall recreational quality indicate that there is more to the change effect than measurement error.

A kind of contextual effect that mainly has been studied experimentally, and with other outcome variables, is the interaction between perceptions of different types of sensory stimuli experienced in a specific setting [5,36,37,41,46–50]. The tendency found that annoyance with other factors than noise changes after the airport change may in itself be interpreted as an “overreaction effect”. But the interaction effect between sound and visual stimuli illustrated in the literature was not related to changes in any conditions. An alternative explanation could be that the different noise exposure levels at t_1_ and t_2_ differently influence the perception of the visual stimuli. The resulting difference in annoyance with the various area conditions would still be the effect of changing noise levels, but would not necessarily in itself be an “overreaction effect.”

The interaction between the perception of sound and other area conditions could also offer an explanation why the change in aircraft noise annoyance following abrupt changes in noise exposure was not very well predicted by exposure-annoyance relationships derived from a steady-state situation [22,23]. One reason might be that the changes in noise exposure affect a broader set of experiential dimensions that interact with noise annoyance. However, we propose that the effect of change is something else or more than just aligning the perceptions of different area conditions as in a steady state situation. According to cognitive consistency theories, the process of reaching a verdict over a situation is a complex dynamic process, where the various components and the general impression mutually influence each other [51]. As suggested in the introduction, the perception of change itself, to the better or the worse, may be an overarching experience that influences both the perception of the various area components and the general impression of the area. A relationship between noise annoyance and perceived overall recreational quality of the area was also demonstrated in both study areas. Both interaction with other area components, the general impression of change and changed area quality may influence aircraft noise annoyance, and thus strengthen the effect of the actual change in noise exposure. Cognitive consistency theories offer a plausible explanation of the underlying perceptual mechanisms of the change effect, in accordance with our findings. Our results and this interpretation of the underlying mechanisms are also compatible with the change in modifying variables explanation of the change effect. This explanation suggests that variables modifying the exposure-annoyance relationship may become more positive when noise exposure decreases and more negative when it increases, thus changing the exposure-annoyance relationship [34].

Significant differences in annoyance with other area conditions between t_1_ and t_2_ were only found in one of the study areas, however. The change in overall aircraft noise exposure was larger at Bygdøy, at the old airport, than in Romeriksåsen, near the new airport. The change in proportions annoyed by sound from aircraft was accordingly larger at Bygdøy than in Romeriksåsen. The weak, but insignificant tendency toward increased proportions annoyed by other factors in Romeriksåsen indicates that there could possibly have been a similar effect in the area near the new airport if the change in noise exposure had been larger. Another factor influencing the results might be the overall level of perceived area problems (Figures 3 and 4), which was higher at Bygdøy at t_1_ than in Romeriksåsen, where other aspects than aircraft noise were almost not a problem. It might be that the overall problems were too small, or too rarely met in the vast area, that the perception of them could be influenced by the changed noise conditions.

### 4.4. Management Implications and Directions for Future Research

The indicated interaction between annoyance with aircraft noise and with other area factors, as well as the relationship between noise annoyance and perceived overall recreational quality of the area, imply that aircraft noise may affect perceived benefits more broadly than interfering with natural quiet and causing noise annoyance. This is in line with the findings in the experimental studies [37–39] that found noise to influence a whole range of visual outcomes, as well as affect. The results point to the need of examining a broader range of experiential effects of noise exposure than noise annoyance alone, especially in regard to abrupt changes in noise exposure levels. The area problems included in this study, in addition to aircraft noise, were chosen because they were relevant in the special study areas. Other problems may be more important in other areas. It would be useful for managers to know not only what problems they have to deal with, one by one, but how the perception of different area problems possibly interact in influencing the perception of the recreational qualities of the area.

## 5. Conclusions

In planning new air routes, it is essential for the authorities to have sufficient knowledge of the effects of aircraft noise, not only on residential areas, but on outdoor recreational areas as well. This study indicates that changed noise exposure may have experiential implications beyond the effect on degree of noise annoyance. It may influence the experience of other single potentially adverse aspects of the environment, and the perception of the overall recreational qualities of the area. The consequence may be altered opportunities (increased or decreased, depending on the direction of change) for recreational goal attainment in the area. Regarding the effect of changed noise exposure on the perception of other area aspects, the significance of the initial noise exposure levels, the amount of change, and the direction of chance should be further studied. More research is warranted to address the question about how the perceptions of different environmental factors interact in forming the outdoor recreational experience.

## Figures and Tables

**Table 1.** Aircraft noise levels, Bygdøy and Romeriksåsen, 1998 and 1999 1.

	**Bygdøy**		**Romeriksåsen**	
	1998	1999	1998	1999
Aircraft L_Aeq_^2^ [dB]	67	49	43	45
Proportion of time over 55 dB [%]	11	3	0.4	1
Proportion of time aircraft can be heard [%]	37	15	12	27
N	962	962	290	705

1 Mean values for individual visitors’ exposure. Data from field studies. Measurement period: Daytime at weekends. For Romeriksåsen, the exposure levels are averaged over three measurement sites. All differences between 1998 and 1999 are significant at *p* < 0.001.

2 L_Aeq_ = A-weighted equivalent sound levels.

**Table 2.** Perceived changes in recreational quality of the areas at t_2_. Percentages.

	**Bygdøy**	**Romeriksåsen**
Better	51	10
Both better and worse	1	4
Worse	8	38
No difference	36	47
Not sure	4	2

Total	100	100
*n*	591	455

**Table 3.** Bygdøy. Perceived better recreational quality at t_2_ dependent on annoyance with aircraft noise at t_1_. Percentages. (*n*=591, visitors both years) 1.

	**Annoyed by aircraft noise at t**_**1**_	
	**not or slightly**	**rather or very**
Recreational quality better at t_2_	43	62
*n*	301	290

1 Chi-Square = 23.42 with 1df, *p* < 0.001

**Table 4.** Romeriksåsen. Perceived worse recreational quality at t_2_ dependent on annoyance with aircraft noise at t_2_. Percentages. (*n* = 455, visitors to the same part of Romeriksåsen both years) 2.

	**Annoyed by aircraft noise at t**_**2**_	
	**not or slightly**	**rather or very**
Recreational quality worse at t_2_	19	70
n	259	196

2 Chi-Square = 11.97 with 1df, *p* < 0.001
